# A 3.7 Mb Deletion Encompassing ZEB2 Causes a Novel Polled and Multisystemic Syndrome in the Progeny of a Somatic Mosaic Bull

**DOI:** 10.1371/journal.pone.0049084

**Published:** 2012-11-09

**Authors:** Aurélien Capitan, Aurélie Allais-Bonnet, Alain Pinton, Brigitte Marquant-Le Guienne, Daniel Le Bourhis, Cécile Grohs, Stéphan Bouet, Laëtitia Clément, Laura Salas-Cortes, Eric Venot, Stéphane Chaffaux, Bernard Weiss, Arnaud Delpeuch, Guy Noé, Marie-Noëlle Rossignol, Sarah Barbey, Dominique Dozias, Emilie Cobo, Harmonie Barasc, Aurélie Auguste, Maëlle Pannetier, Marie-Christine Deloche, Emeline Lhuilier, Olivier Bouchez, Diane Esquerré, Gérald Salin, Christophe Klopp, Cécile Donnadieu, Céline Chantry-Darmon, Hélène Hayes, Yves Gallard, Claire Ponsart, Didier Boichard, Eric Pailhoux

**Affiliations:** 1 INRA, UMR1313 Génétique Animale et Biologie Intégrative, Jouy-en-Josas, France; 2 UNCEIA, Service Génétique, Paris, France; 3 INRA, UMR 1198 Biologie du Développement et Reproduction, Jouy-en-Josas, France; 4 INRA-ENVT, UMR 444 Génétique Cellulaire, Toulouse, France; 5 UNCEIA, Département Recherche et Développement, Maisons-Alfort, France; 6 Institut de l’Elevage, Département Génétique, Identification, Phénotypes et Systèmes d'Information en Elevage, Limoges, France; 7 Labogena, Jouy-en-Josas, France; 8 INRA, UE0326 Domaine expérimental du Pin-au-Haras, Exmes, France; 9 GeT-PlaGe, Genotoul, Castanet-Tolosan, France; 10 INRA, UMR444 Génétique Cellulaire, Castanet-Tolosan, France; 11 INRA, Plateforme bioinformatique Genotoul, UR875 Biométrie et Intelligence Artificielle, Castanet-Tolosan, France; The University of Tennessee Health Science Center, United States of America

## Abstract

Polled and Multisystemic Syndrome (PMS) is a novel developmental disorder occurring in the progeny of a single bull. Its clinical spectrum includes polledness (complete agenesis of horns), facial dysmorphism, growth delay, chronic diarrhea, premature ovarian failure, and variable neurological and cardiac anomalies. PMS is also characterized by a deviation of the sex-ratio, suggesting male lethality during pregnancy. Using Mendelian error mapping and whole-genome sequencing, we identified a 3.7 Mb deletion on the paternal bovine chromosome 2 encompassing *ARHGAP15*, *GTDC1* and *ZEB2* genes. We then produced control and affected 90-day old fetuses to characterize this syndrome by histological and expression analyses. Compared to wild type individuals, affected animals showed a decreased expression of the three deleted genes. Based on a comparison with human Mowat-Wilson syndrome, we suggest that deletion of *ZEB2*, is responsible for most of the effects of the mutation. Finally sperm-FISH, embryo genotyping and analysis of reproduction records confirmed somatic mosaicism in the founder bull and male-specific lethality during the first third of gestation. In conclusion, we identified a novel locus involved in bovid horn ontogenesis and suggest that epithelial-to-mesenchymal transition plays a critical role in horn bud differentiation. We also provide new insights into the pathogenicity of *ZEB2* loss of heterozygosity in bovine and humans and describe the first case of male-specific lethality associated with an autosomal locus in a non-murine mammalian species. This result sets PMS as a unique model to study sex-specific gene expression/regulation.

## Introduction

Cranial appendages are recently acquired structures in the evolution of Mammals. Successive environmental and behavioral changes have favored the emergence of diverse forms in Ruminantia and extinct related groups, among which the following four continue to exist in present-day species: antlers in cervids, horns in bovids, ossicones in giraffids and pronghorns in antilocaprids [1]–[4]. Each of these structures, which start to develop only after birth, represents a valuable model to investigate cell differentiation and reciprocal interactions between tissues during organogenesis. Their study could lead to important applications in biomedical fields such as skin regeneration, bone cancer, and osteoporosis (for review see [5]). They could also provide new insights into sex-specific gene expression/regulation as suggested by morphological differences between genders and by the association of horn agenesis and intersexuality in the goat Polled Intersex Syndrome (PIS).

Whereas the regeneration of antlers in cervids has become a major research topic, the development of horns in bovids has received comparatively little attention. However, the genetic mapping of hornless phenotypes segregating in domestic species represents a unique opportunity to unequivocally isolate genes involved in horn ontogenesis. To date, four loci have been analyzed: i) the mutation responsible for the goat PIS, a 11.7 kb deletion, has been shown to affect the transcription of at least three genes: *FOXL2, PISRT1* and *PFOXic* [6], [7]; ii) ovine and bovine polled loci have been mapped to small genome intervals containing respectively *RXFP2* and, *IL10RB, IFNAR2, OLIG1* and *OLIG2* genes but their causative mutations have not yet been published or definitely identified [8]–[10] (and Capitan et al., unpublished data); and iii) recently our group reported a novel type 2 scurs syndrome associated with the loss of TWIST1 heterozygosity [11]. Nevertheless, it is still unclear how these genes belonging to different pathways can cooperate and participate to horn bud differentiation during embryogenesis or horn growth after birth. In an attempt to identify new genes involved in these processes and to gain better insights into horn ontogenesis, we screened the whole French cattle population for new horn development anomalies. Among the numerous records, one particular case caught our attention: a Charolais bull (V.), born to horned parents, that never developed normal horns but instead small horny scabs and for which the polled progeny displayed severe additional symptoms.

Here, we report the clinical description of this new syndrome and the identification of the causative 3.7 Mb deletion. In addition, we present unique histological and gene expression data on bovine horn bud differentiation during embryogenesis and we suggest that epithelial-to-mesenchymal transition plays a critical role in this process. Finally, we provide new insights into *ZEB2* gene function in cattle and humans and describe a rare case of dominant male-specific lethality associated with an autosomal mutation.

## Results and Discussion

### Analysis of Genetic Inheritance

According to breeders reports, V. mated with horned cows sired a total of 76 progeny, consisting in 31 horned females, 29 horned males, 14 polled females and only two polled males. In contrast to what is observed with the regular polled phenotype [12], [13], the gender distribution of this bull’s progeny shows significant differences (chi-square = 6.71, p<0.01) and is incompatible with simple monogenic autosomal dominant inheritance (chi-square = 29.36, p<0.0001). Since the numbers of polled males versus polled females differ with a ratio clearly in favor of females but the numbers of horned males and females are equivalent, we assume that most polled males died during gestation. In addition, we assume that inheritance follows a monogenic autosomal dominant pattern with paternal mosaicism since the clinical course of V. is mild compared to its progeny.

### Clinical Examination

**Figure 1:**
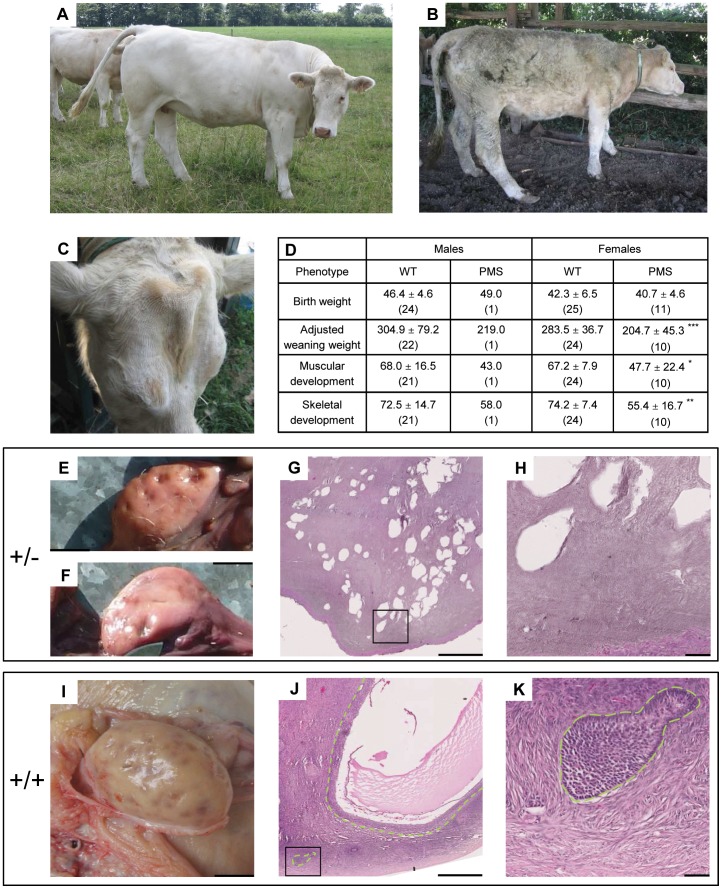
Clinical features of Polled and Multisystemic Syndrome. (A) Two-and-half-year old wild-type heifer that was mechanically dehorned when approximately one-year old. (B) Two-and-half-year old affected heifer. Note the slender build, the shaggy hair coat demonstrating the bad health condition, and the hypotonia of hind limbs. (C) Upper part of the skull of the same affected heifer. Note the absence of corneous growth, the ridge-shaped extra bone deposition along the frontal suture and the narrowness of the muzzle insertion. (D) On-farm performance testing statistics of affected (PMS) and wild-type half-sibs. Values expressed as: means ± standard deviation (number of observations). *p<0.05, **p<0.01 and ***p<0.001 versus wild-type half-sisters (Welch’s t-test). Weaning corresponds to 210 days of age. (E and F) Ovaries of the affected (+/−) heifer displayed in (B) and (C). (I) Ovary of a wild-type (+/+) matched control. (G and H, and J and K) Histological analyses of the ovaries displayed in (E) and (I) respectively. (H) and (K) are higher magnifications (X5.5) of (G) and (J). Note the numerous large lacunae surrounded by connective tissue and the absence of follicles in the ovary from the affected heifer. Follicles are surrounded with a green dotted line in the photography of the wild-type ovary. Scale bars represent 1 cm in (F), (E) and (I); 500 µm in (G) and (J); and 50 µm in (H) and (K).

In contrast to V., its polled progeny was characterized by complete horn agenesis; facial dysmorphism with frontal bossing and a narrow muzzle (Figure 1A–1C); variable neurological disorders including reduced and delayed response to environmental stimuli, hypotonia, apathy, anorexia, and, in one case, ataxia; postnatal growth retardation (Figure 1D); chronic diarrhea; and female reproductive anomalies (no heat signs for the nine females reaching sexual maturity). Clinical examination of the two affected females still alive at the time of the study showed a normal reproductive system except for very small ovaries, pale vulvar vestibular mucosa and no cervical mucus. Moreover, progesterone concentrations were low (<1 ng/ml) indicating acyclicity. After one of these females died, visual inspection of its ovaries showed a few corpus albicans demonstrating past sporadic ovulation (Figure 1E and 1F). Surprisingly, compared to matched controls, histological analysis detected no follicle (Figure 1G–1K) indicating that premature ovarian failure (POF) occurred early in the reproductive life of this animal. Its autopsy also revealed a right heart ventricular hypertrophy without structural heart malformations and a jejunal volvulus with focal hemorrhagic enteritis (Figure S1), most probably due to hyperperistaltism associated with chronic diarrhea. Given the range of symptoms associated with horn agenesis, this new condition was named Polled and Multisystemic Syndrome (PMS).

### Mapping and Identification of the Causative Mutation

**Figure 2:**
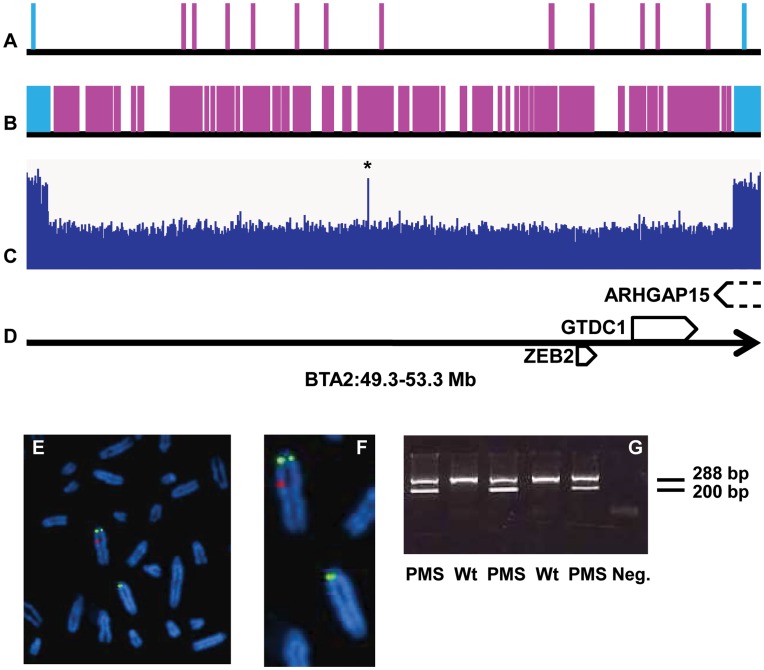
Mapping and characterization of the causative mutation for PMS syndrome. (A) and (B) Results of Mendelian error mapping using the Illumina 50 K and 777 K SNP beadchips, respectively. Markers displaying Mendelian errors between at least one PMS heifer and her sire are represented in purple whereas markers for which at least one of the three PMS animals is heterozygous are represented in blue. Other markers are not represented. (C) Plot of the whole-genome sequencing read depth coverage on the same region. *: artifact due to a local error in genome assembly. (D) Gene content of the region. (E) FISH-mapping with BAC clones located in the deleted region (labeled in red) and in the juxtacentromeric region of BTA2 (labeled in green) on fibroblasts of a PMS animal. (F) Magnification of (E) showing normal (above) and deleted (below) BTA2 chromosomes. (G) Genotyping of PMS using a three-primer PCR system (see methods). Neg.: negative control.

To investigate the molecular basis of PMS, we genotyped V., 19 unaffected progeny, three affected daughters and their dams with the Illumina bovine 50 K Single Nucleotide Polymorphism (SNP) beadchip. To identify putative large deletions, Mendelian error detection was performed. Compared to control animals, the affected animals had many more Mendelian errors, mainly clustered within a 2.8 Mb region on bovine chromosome 2 (BTA2, Figure 2A). Haplotype reconstruction in this region revealed that while their unaffected half-sib had inherited one of the two haplotypes carried by V., the three affected heifers were hemizygous for their maternal haplotypes. To refine the localization of the deletion breakpoints, these heifers and V. were genotyped with the Illumina bovine 777 K SNP beadchip (Figure 2B) and the complete genome of one heifer was sequenced using 100-bp paired-end reads. We identified a 3,708,143-bp deletion and a 4-bp insertion (ACAT) between positions 49,422,588 and 53,130,732 bp on BTA2, according to the UMD3.1 bovine genome assembly [14] (Figure 2C). This deletion was further confirmed by Sanger sequencing, fluorescence in situ hybridization (FISH) and PCR electrophoresis (Figure 2E–2G). Taken together, these results demonstrate that V. is mosaic for a large somatic deletion on BTA2, which is responsible for PMS.

This deleted region contains the genes *ZEB2*, *GTDC1* and the last exon of *ARHGAP15* (Figure 2D).

*GTDC1* (glycosyltransferase-like domain containing 1) [15] encodes a protein of unknown function which shares a conserved domain with mannosyltransferase III and V in human, two proteins involved in the synthesis of lipid linked oligosaccharides [16]. *ARHGAP15* encodes Rho GTPase activating protein 15, a master regulator of neutrophil functions [17]. Studies in mice have shown that ARHGAP15-deficient animals are viable and fertile [17]. Finally, *ZEB2* (zinc finger E-box binding homeobox 2) encodes a zinc finger nuclear transcription factor which is involved in numerous processes. Interestingly, in humans, loss of *ZEB2* heterozygosity is responsible for Mowat-Wilson syndrome [18] (MWS; OMIM #235730), a multiple congenital anomaly syndrome sharing strong similarities with PMS [19], [20]. MWS and PMS are characterized by facial dysmorphism, postnatal growth retardation, congenital heart defects and neurological disorders. They also include antagonistic intestinal disorders consisting in chronic diarrhea in cattle and chronic constipation in humans (Hirschsprung disease; HSCR). However, a recently reported case of a patient with both HSCR and MWS presented a supernumerary intestinal muscle coat and abnormal gut motility, and developed chronic diarrhea after surgical resection of the aganglionic colon [21], thus reconciling both observations.

### Characterization of PMS Symptoms in Fetuses

**Figure 3:**
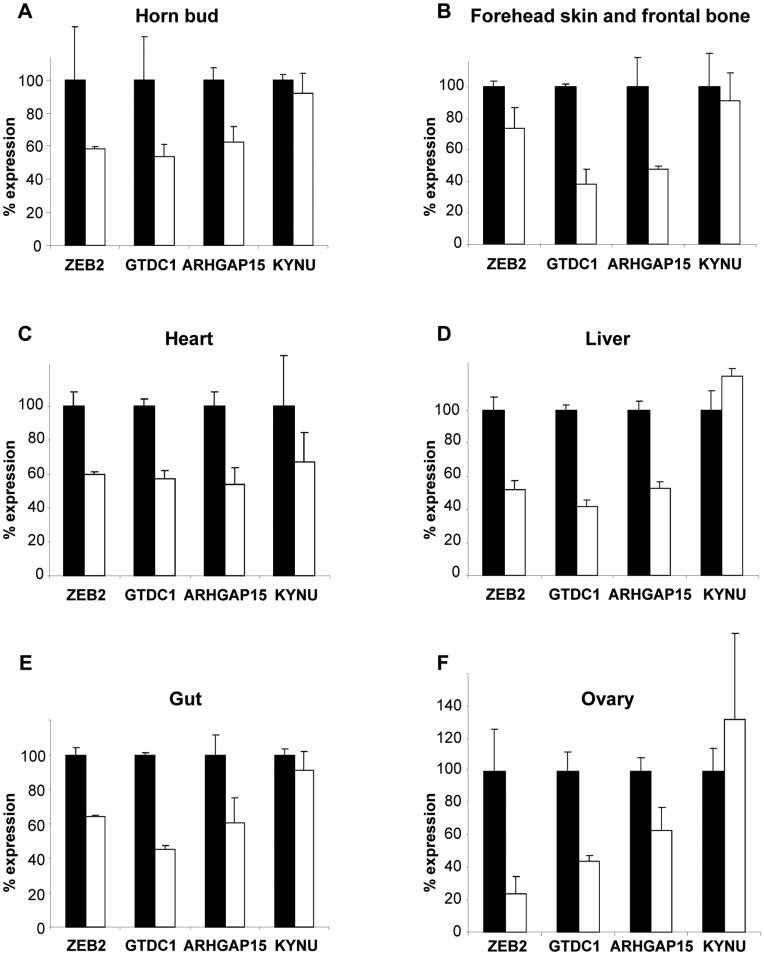
Real-time PCR expression analyzes of the deleted genes in different affected tissues at 90 dpc. *ZEB2*, *GTDC1*, *ARHGAP15* (exon13), and *KYNU* (absent from the deleted fragment) expression is measured in various tissues from wild type (+/+; black histograms) or mutant (+/−; white histograms) fetuses.

To investigate the consequence of the deletion, we produced PMS and wild-type (wt) female fetuses at 90 dpc (days *post-coïtum*), corresponding to when the horn bud becomes visible [22]. Using Reverse Transcription quantitative PCR (RT-qPCR), we analyzed the gene expression of *ZEB2*, *GTDC1*, *ARHGAP15* and *KYNU* (the closest non-deleted gene) in tissues with defects possibly originating during development and in asymptomatic tissues (Figure 3). Whereas no difference was found in expression levels of the *KYNU* gene between PMS and wt tissues, expression levels of the deleted genes were approximately reduced by half in all tested tissues in the hemizygous fetuses suggesting that a reduced amount of mRNA of at least one of these is responsible for PMS. In addition, we investigated the regulation of *ZEB2* gene expression by its natural antisense transcript [23] and found that loss of heterozygosity had no consequence on expression of the remaining allele of this gene (Figure S2). Although our results do not point to one precise gene, the decreased expression of *ZEB2* stands as the most probable cause of the common features between PMS and MWS, since patients with truncating mutations in *ZEB2* or with large deletions encompassing *ZEB2*, *GTDC1* and *ARHGAP15* do not show any significant phenotypic difference [24]–[26].

Next, we focused on the characterization of the three features that are specific to PMS, i.e. horn agenesis, female infertility and male-specific lethality.

**Figure 4:**
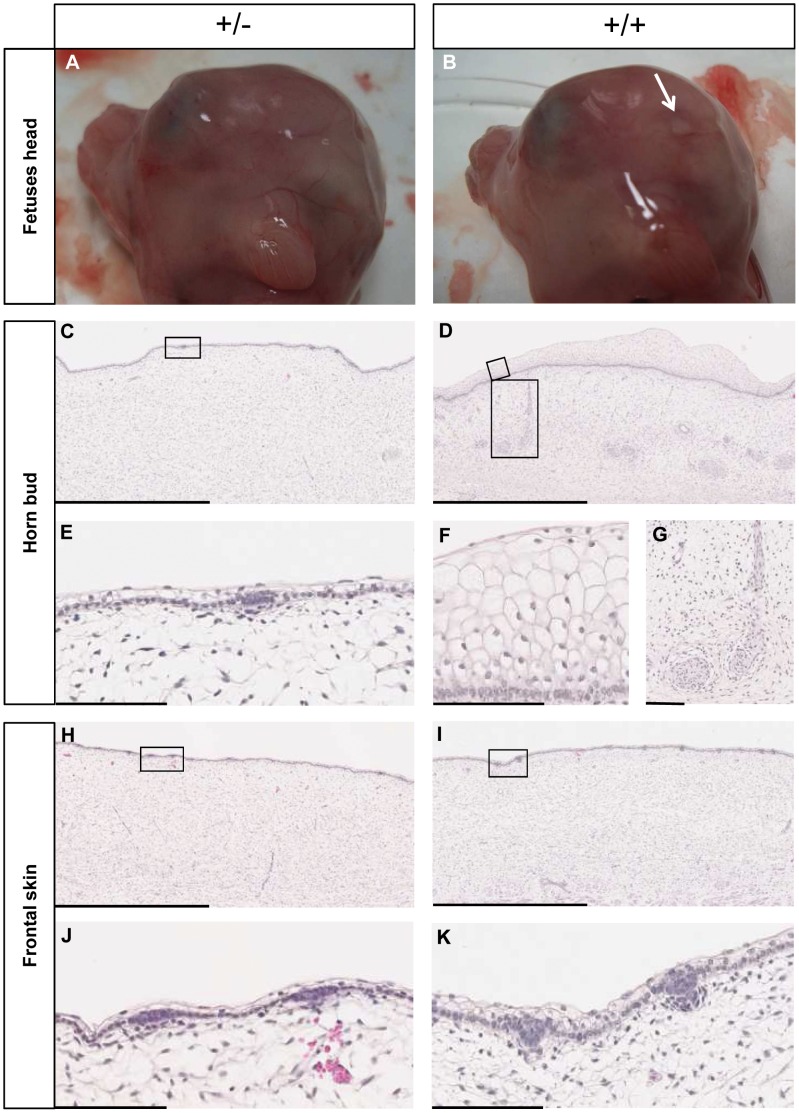
Histological analyses of wild-type (+/+) and PMS (+/−) horn bud and forehead skin. (A) and (B) Head of PMS (+/−) and wt (+/+) fetuses. Horn bud of wt fetus is indicated by an arrow. (C) and (D) Histological sections of the “horn bud” of PMS and wt fetuses respectively. (E) Magnification (X10) of (C) showing one hair follicle primordium. (F) and (G) Magnifications (X10 and X3 respectively) of (D) showing respectively keratinizing epidermal cells and clusters of dermal cells displaying glandular/ductal differentiation. (H) and (I) Histological sections of the forehead skin of PMS and wt fetuses respectively. (J) and (K) Magnifications (X10) of (H) and (I) showing hair follicles primordial; note the slight difference between PMS and wt genotypes; statistical analysis also showed a significant difference in epidermis thickness: 15.8±3.0 µm in PMS vs 22.1±3.7 µm in wt; p-value = 2.3e-28 (Welch’s t-test). Scale bars in (C), (D), (H) and (I) represent 1 mm whereas scale bars in (E), (F), (G), (J) and (K) represent 100 µm.

Visual examination of control and PMS fetuses showed that only PMS fetuses had no horn buds (Figure 4A and 4B). Serial histological sections confirmed that “horn buds” and neighboring skin were identical in PMS fetuses. In contrast, horn buds from controls showed supernumerary layers of vacuolated keratinocytes and clusters of dermal cells displaying glandular/ductal differentiation (Figure 4C–4G). Intriguingly, whereas epidermis starts to produce the first layers of the future keratin sheath, no evidence of osteoblast or chondroblast differentiation was found in the wt horn bud dermis at this stage. On the contrary, the presence of skin glands’ primordia, structures not detectable in frontal skin dermis at this stage, suggests that differentiation of horn bud dermis occurs earlier but is similar to that of the rest of the skin. Thus, initiation of dermis ossification to form the horn bony core may occur later in fetal life or after birth. These observations confirm that the wt horn bud starts to differentiate long before birth and that the cause of polledness in PMS is an impaired differentiation of the horn bud. Interestingly, a careful examination of histological sections revealed that differentiation of the forehead skin was also delayed in PMS fetuses compared to controls with a reduced hair follicle germ size and significantly thinner epidermis (Figure 4H–4K). This observation is consistent with the sparse fine hair observed in MWS infants [19], [27] suggesting that *ZEB2* may be involved in hair follicle and horn bud differentiation. This assumption is also supported by the fact that PMS and bovine type 2 scurs, a syndrome associated with *TWIST1* loss of heterozygosity [11] share a similar phenotype i.e. horn defects with frontal bossing. Indeed, *TWIST1* and *ZEB2* are two of the few master regulators of epithelial-to-mesenchymal transition (EMT), an important developmental process in which new mesenchymal tissue is locally generated from epithelia [28]. EMT has a fundamental role throughout evolution in generating complex body patterns [29]. Interestingly, transcription profile analyses of horn buds from regular polled and horned newborn calves also support postnatal EMT [30]. Taken together, these results not only suggest an essential role of *TWIST1* and *ZEB2* in horn bud differentiation but also of EMT in both horn bud differentiation and horn growth.

**Figure 5:**
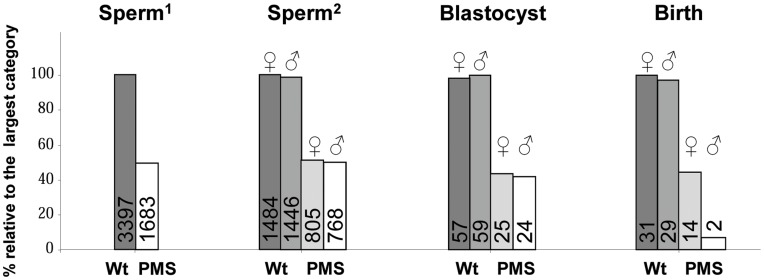
Distribution of PMS at different developmental stages in V.’s progeny. Histograms represent percentages relative to the largest category at each developmental stage. The numbers are indicated on each histogram. (1) and (2) results of the first and second sperm-FISH experiments (see methods). Blastocysts correspond to 7 dpc embryos.

In cow ovarian development, germ cell meiosis ends around 90 dpc [31], a stage at which, we detected no histological difference between PMS and control ovaries using haematoxylin and eosin staining (Figure S3A and S3B) or immuno-histological staining with antibodies against VASA, γH2A and FOXL2 proteins (three markers of germ cells; not shown). RT-qPCR did not reveal any notable difference between the case and control tissues in the expression of meiotic genes (*STRA8* and *SYCP1*), of genes involved in the early stage of follicle formation (*SOHLH1* and *FIGLα*) or of the somatic cell specific gene *FOXL2* (Figure 5C). Cell proliferation was not affected, in regard to *KI67* expression (Figure S3C). Thus, the adult PMS ovarian phenotype is neither due to defective germ cell migration, contrary to what is observed in Drosophila haploinsufficient for *ZFH-1* [32], the ortholog of *ZEB2*, nor to meiotic failure. It is more likely due to a problem during follicle formation or to massive follicular atresia around and after birth. Since many members of the *TGFβ* family are involved in follicle formation, follicle development and POF [33], [34], the decreased expression of *ZEB2*, encoding a Smad interacting protein, remains the most probable cause for POF associated with this syndrome. To date, pubertal development in MWS patients has been poorly studied [19] except for a unique female patient who displayed delayed puberty and inconsistent menarche [35], two symptoms associated with POF. Thus, based on these results, investigating this particular feature in MWS patients would be very interesting.

Although hundreds of autosomal genes undergoing epigenetic regulation by sex chromosomes have been reported [36], [37], very few cases of partial to complete male-specific lethality have been described in mice or mammals [38]–[41]. To confirm that the lack of PMS males observed at birth is due to male-specific lethality and not to another cause, we estimated the number of carriers of the causative deletion at two prenatal stages (in semen and in 7-day blastocysts produced *in vitro* with V.’s semen) and compared the results with observations at birth (Figure 5). We found a similar ratio between phenotypic and gender categories at the different developmental stages except for “males at birth” (chi-square = 2.10, 8.93, and 11.56, p<0.20, <0.01 and <0.001 for goodness of fit test between distributions in sperm and blastocyst, blastocyst and birth and, sperm and birth conditions respectively). These results indicate that male and female PMS spermatozoids have an identical fertilizing power and that most of the PMS males die between 8 dpc and birth (Figure 5). We then analyzed the reproduction records of the cows inseminated with V.’s semen to refine this time interval assuming that part of the unsuccessful inseminations results from male embryonic lethality. We determined that the maximal gestation length for dead male PMS conceptuses was below 88 days (see methods). Thus, most of the PMS males died during the first third of the gestation phase, a critical period during which the placenta and all major organs are formed. Interestingly, El-Kasti *et al.* [42] have recently reported a novel transgenic rodent model in which deletion of an enhancer of *ZEB2*, located 1.2 Mb upstream of this gene, results in an autosomal-dominant phenotype consisting of a severe attenuation of postnatal kidney development in males. Intriguingly, other aspects of embryonic and neonatal development were unaffected, supporting the fact that expression of *ZEB2* may be influenced by gender in specific tissues at particular developmental stages through the action of specific regulatory elements. Contrary to our results on PMS, analysis of the sex-ratio of four male and five female MWS patients with deletions encompassing *ZEB2*, *GTDC1* and *ARHGAP15* showed no significant deviation [43]. However, these human patients carried different deletions and only a few of them encompassed the enhancer of *ZEB2* mentioned above, as is the case for the 3.7 Mb deletion reported in bovine. In addition, recurrent “autosome-to-Y” gene transpositions in mammalian evolution have introduced numerous differences between human and bovine Y-chromosomes [44] that may result in different interactions with autosomal genes. Male-specific lethality may also result from a particular configuration of the PMS causative mutation leading to ectopic expression of surrounding genes. To address this question, PMS male blastocysts will be transferred in recipient cows and their gestation will be carefully analyzed in the future.

Finally, sperm-FISH experiments revealed a high ratio of mutant versus wt spermatozoids (33 and 35%) corresponding to a high level of germline mosaicism (66 to 70%). Severe horn atrophy and clear positive PCR diagnosis on DNA extracted from V. blood (not shown) also support the hypothesis of a high level of whole-body mosaicism in the founder bull, suggesting that the causative deletion occurred in the very first cellular division after fecundation.

In conclusion, we describe a bovine counterpart of human Mowat-Wilson syndrome in the progeny of a somatic mosaic bull. This case is exceptional both because of its probability of occurrence and its clinical features. Indeed, the level of mosaicism in the founder bull was sufficiently low to permit its survival during gestation and a healthy state and, at the same time, sufficiently high for it to be naturally dehorned and thus selected for reproduction. In addition to the main features of human Mowat-Wilson syndrome and to horn agenesis, its affected progeny displayed premature ovarian failure in females and specific lethality of males during pregnancy, thus preventing this syndrome to pass on to the next generation. Here, we identified a 3.7 Mb causative deletion encompassing three genes, and provide strong evidence for the decreased expression of *ZEB2* being responsible for most of the symptoms. We present unique histological and gene expression data on bovine horn bud differentiation during embryogenesis and suggest that *TWIST1*and *ZEB2* genes and epithelial-to-mesenchymal transition play a critical role in this process. We also provide new insights into the pathogenicity of *ZEB2* loss of heterozygosity in cattle and humans and suggest that pubertal development should be examined in Mowat-Wilson female patients. Finally we describe the first case of male-specific lethality associated with an autosomal locus in a non-murine mammalian species. Our results open new research avenues to better understand bovine horn ontogenesis, premature ovarian failure and sex-specific gene expression/regulation.

## Materials and Methods

### Ethics Statement

Experiments reported in this work comply with the French National Institute for Agricultural Research (INRA) ethical guidelines. The protocol has been approved by the Division of Social Cohesion and Protection of Populations from the Orne Department (DDCSPP 61) and Aurélien Capitan is recipient of an official authorization for animal experimentation from the DDCSPP 61.

### Animals, Clinical Examination and Sampling

At the beginning of the study, only two PMS-affected heifers were still alive. Both heifers were visually inspected at 906 and 918 days of age. Their reproductive tract was examined using a vaginoscope and transrectal palpation. Plasma progesterone was measured at day 0 and 12 by ELISA (OVUCHECK kit, BIOVET Inc) according to the manufacturer’s recommendations. Numbers of Cryptosporidia cysts and nematode eggs per gram of feces were determined by modified Ziehl-Neelsen and modified McMaster methods respectively [45], [46], and were found normal. In addition, herd health records were checked for negative tests for Bovine Viral Diarrhea virus and paratuberculosis to confirm the endogenous origin of chronic diarrhea. Both heifers died a natural death and on-field autopsy of one heifer was performed (at 935 days). Organs from the thoracic and abdominal cavities were visually examined and the ovaries sampled. PMS clinical spectrum was also established from breeders and veterinarian reports, and from on-farm performance records. Estimated age for female sexual maturity in the Charolais breed is 426+/−42 days [47]. Reproduction records (artificial insemination and calving dates) of the cows inseminated with V.’s semen were obtained from the French national database for genetic evaluation. V. has been used as a natural service bull in one farm and as an artificial insemination (AI) bull in three French and two Austrian farms. Sixty-one AI were performed in France: 26 produced at least one calf and 35 were recorded as unsuccessful. Since part of these failures is due to embryonic male lethality, we used these data to determine the maximal gestation length of dead PMS conceptuses by calculating the time-length between the failed AI and another AI (i.e. another observed heating) or between the failed AI and the next calving, subtracting the average gestation (285 days) and sexual cycle (21 days) lengths. Most of these cows were kept with natural service bulls one or two months after AI. Calculations used 20 unsuccessful AI records since we discarded one AI performed after superovulation, six performed one day before another AI and eight performed on low-fertility females (i.e. with three or more consecutive AI failures).

DNA from two PMS-affected heifers and their dams was extracted from blood using the Wizard® Genomic DNA purification Kit (Promega). DNA samples from V., 19 unaffected progeny (7 males, 12 females) and 11 of their dams and one PMS heifer and her dam, previously collected for parentage testing and extracted from blood and/or ear biopsies, were available from INRA Labogena platform.

Case and control embryos and fetuses were produced as previously described [48]. Briefly, oocytes from slaughterhouse ovaries were *in vitro* matured, fertilized with V.’s semen, biopsied on day 7 and frozen. After preimplantation diagnosis, PMS and wt female embryos were implanted in cull cows. Pregnant cows were slaughtered on day 90 (by stunning and subsequent bleeding) and the dead fetuses recovered from their genital tracts. From each fetus, the right horn bud and frontal bone, right forehead skin and frontal bone, heart, liver, gut, and right ovary were collected for expression studies whereas the left horn bud and frontal bone, left forehead skin and frontal bone and left ovary were collected for histological analyses.

### Mapping of the Causative Mutation

SNP genotyping was done using the BovineSNP50 and BovineHD Beadchips (Illumina Inc.). Mendelian error mapping used in-house software searching for the SNP for which the bull and its progeny showed opposite homozygous genotypes. Chromosome 2 haplotyping was performed using MERLIN software [49]. Marker order and map distances were based on the UMD3.1 bovine sequence assembly. To avoid any bias, genotypes showing Mendelian errors were set as missing before phasing and complemented thereafter.

### Identification of the Causative Mutation

A paired-end library with a 250-bp insert size was generated for one PMS-affected heifer using the Illumina TruSeq DNA Sample Prep Kit. The library was quantified using QPCRLibrary Quantification Kit (Agilent), controlled on a High Sensitivity DNA Chip (Agilent) and sequenced on two HiSeq 2000 lanes (Illumina) with Illumina TruSeq V3 Kit (200 cycles). The 100-bp reads were mapped on the UMD3.1 bovine sequence assembly using the BWA tool [50]. The deletion breakpoints were identified using the Integrative Genomics Viewer [51] and selecting reads with unmapped paired-ends or discordant insert sizes in the intervals determined after the mapping results. The mutation was characterized by aligning the unmapped paired-end reads altogether and BLATing this local sequence assembly to the bovine genome with the University of California, Santa Cruz (UCSC) genome browser (http://genome.ucsc.edu/). Then, a 200-bp fragment spanning the mutation was PCR-amplified with the PMS-F (TTGGGGAGAAAATGTGATGC) and PMS-Del-R (ATTTTTCCTGGCAATCCTGA) primers using the Go-Taq Flexi DNA Polymerase (Promega) according to the manufacturer’s instructions. This amplicon was purified on a MultiScreen PCR96 Filter Plate (Millipore) and bidirectionally sequenced by Qiagen (Hilden, Germany) using conventional Sanger sequencing. Finally the whole pedigree was genotyped using a 3-primer PCR system (PMS-F, PMS-Del-R and PMS-R: AAAATTGGCAACGTCCTCTG) under the same conditions. PCR products were visualized on a 2% agarose gel.

### Assessment of the Gene Content of the Deleted Region

The gene content of the deleted region was assessed based on the gene annotation available for the UMD3.1 genome assembly. Genes corresponding to retrotransposed pseudogenes or expressed sequence tags absent from this region in other mammalian species (cat, dog, horse, human, mouse, rabbit, rat, sheep) and showing a higher BLAT identity score in other positions on the bovine genome were discarded.

### FISH

Skin biopsies were obtained from one PMS-affected and one wild-type female fetus. Fibroblast cultures and metaphases were obtained according to Ducos *et al.* [52]. BLAST was used to identify clone end sequences in the deleted region and in the juxtacentromeric region of BTA2, as a reference (http://blast.ncbi.nlm.nih.gov/Blast.cgi). Two INRA BAC clones [53] were selected: 227E10 (chr2:50,399,778-50,500,892; UMD3.1) and 421B09 (chr2:4,870,080-4,989,775; UMD3.1). Dual-color FISH [54] was performed with DNA extracted from BAC 227E10 labeled with Alexa 594 (red) and 421B09 with Alexa 488 (green) (Molecular Probes).

### Sperm-FISH Analysis

Spermatozoa decondensation was carried out according to Hassanane *et al.* [55] with optimal decondensation reached in 3 to 4 minutes. Two sperm-FISH experiments were carried out according to Pinton *et al.* [56], one with BAC clones 227E10 and 421B09 revealed respectively with Alexa 594 and Alexa 488 and one with BAC clones 981H08 (for chromosome Y) and 227E10 revealed respectively by Alexa 488 and Alexa 594. The slides were observed under a Zeiss Axioskop microscope fitted with a triple bandpass filter and only sperm heads exhibiting high intensity signals were scored.

### Embryo Genotyping

On day 7, embryos were biopsied (5 to 10 cells) and whole-biopsy amplification of genomic DNA was performed using Repli-g® Mini Kit (Qiagen). Sex determination was done by PCR-electrophoresis (UNCEIA Sexing Kit, UNCEIA, Paris, France). The PMS status was determined using the 3-primer PCR system described above. In case of low PCR amplification or ambiguous results, PCR were repeated twice. Finally, 165 of 174 embryos, with both accurate sex and PMS status, were available to study the distribution of PMS in V.’s progeny.

### Quantitative RT-PCR

RNA was extracted using the RNeasy Mini kit (Qiagen). Super-Script II (Invitrogen) was used to synthesize cDNA from 2 µg of total RNA isolated from 90 dpc fetal tissues (horn bud and frontal bone, forehead skin and frontal bone, heart, liver, gut, and ovary). Two distinct fetuses were used for both genotypes. Bovine gene sequences were obtained from UCSC genome browser and PCR primers (Table S1) were designed using Primer Express Software for Real-Time PCR 3.0 (Applied Biosystems). Primer efficiency and specificity were evaluated on bovine genomic DNA. Quantitative PCR was performed in triplicate, using the Absolute Blue SYBR Green ROX mix (Thermo Fisher Scientific) and the StepOnePlus Real-Time PCR system (Applied Biosystems). Results were analyzed with the Qbase software using three appropriate normalizing genes (*ACTB*, *YHWAZ* and *H2AFZ*), as previously described [57]. Gene expression is presented as a percentage of the maximum expression value obtained.

### Histological Preparation

Ovaries from the two-and-half-year old PMS heifer and a two-year old wt heifer were fixed in formol (10%) and tissues from 90 dpc fetuses were fixed in paraformaldehyde (4%) at 4°C. Tissue fragments were dehydrated in a graded ethanol series, cleared with butanol and embedded in paraffin. Microtome sections (7 µm, Leica RM2255) were stained with haematoxylin and eosin (HE). Digital images were obtained with the NanoZoomer 2.0-HT (Hamamatzu).

### Statistical Analyses

A first Pearson’s Chi-squared test was used to test for independence between gender and PMS status in V.’s progeny. Then, goodness-of-fit between PMS distribution in V.’s progeny and autosomal dominant inheritance was tested. Goodness-of-fit between PMS distribution in V.’s semen versus V’s blastocysts, V.’s semen versus V’s progeny and V’s blastocysts versus V’s progeny was also tested. Significant differences between PMS and wt female on-farm performance means were determined with Welch’s t-test. This procedure is more robust against variance heterogeneity than Fisher’s t-test, especially when sample sizes differ. Birth weight, adjusted weaning weight, muscular and skeletal development scores follow normal distributions. Fifty random measurements of forehead epithelium thickness (five on ten histological sections) were done for each of the two PMS and two wt fetuses. The Shapiro-Wilk test was used to test for normal distributions. Then Welch’s t-test was used to determine any significant difference in thickness between PMS and wt groups. For each test, p-values superior to 0.05 were considered as not significant.

## Supporting Information

Figure S1 **Additional illustrations of PMS clinical features.** (A) Two-and-half-year old affected heifer displaying chronic diarrhea. (B) Autopsy of the abdominal cavity showing jejunal volvulus (JV) with focal hemorrhagic enteritis. (C) Heart of the same animal. White line indicates the section plane. (D) Transversal section of PMS heart showing ventricles in partially open position. RVW and LVW indicate respectively right ventricle wall and left ventricle wall.
(TIF)

Figure S2 **Analysis of** ***ZEB2*** **expression regulation by its natural antisense transcript in different affected tissues at 90 dpc using real-time PCR.** Localisation of PCR primers relatively to *ZEB2* gene and its natural antisense transcript (NAT) are indicated by purple arrows. CT: Cycle Thresholds. Note the proportional reduction of *ZEB2* exon 3, *ZEB2* NAT and *ZEB2* intron 1 RNA amounts in PMS versus wild type fetuses in the different organs studied.
(TIF)

Figure S3 **Histological and gene expression analyses of wild-type (+/+) and PMS (+/−) 90 dpc ovaries.** Ovarian histology in +/+ (A) or +/− (B) fetuses at 90 dpc. The green dotted line shows the separation between the cortical (co) and the medulla (m) part of the ovaries. Scale bar represents 50 µm. (C), RT-PCR expression analyses of germ cells (GC) specific markers such as *VASA* and meiotic markers (*STRA8* and *SYCP1*), or genes involved in follicle formation (*SOHLH1* and *FIGα*), in wild type or mutant 90 dpc ovaries. The somatic cell (SC) marker *FOXL2* and proliferation factor (*KI67*) were also studied.
(TIF)

Table S1 **Primers used in RT-qPCR study.**
(DOC)
