# Supplementary figures and images for: A 3.7 Mb Deletion Encompassing ZEB2 Causes a Novel Polled and Multisystemic Syndrome in the Progeny of a Somatic Mosaic Bull
Source: PLoS One. 2012 Nov 9;7(11):e49084. doi: 10.1371/journal.pone.0049084 (PMC3494662; doi:10.1371/journal.pone.0049084)

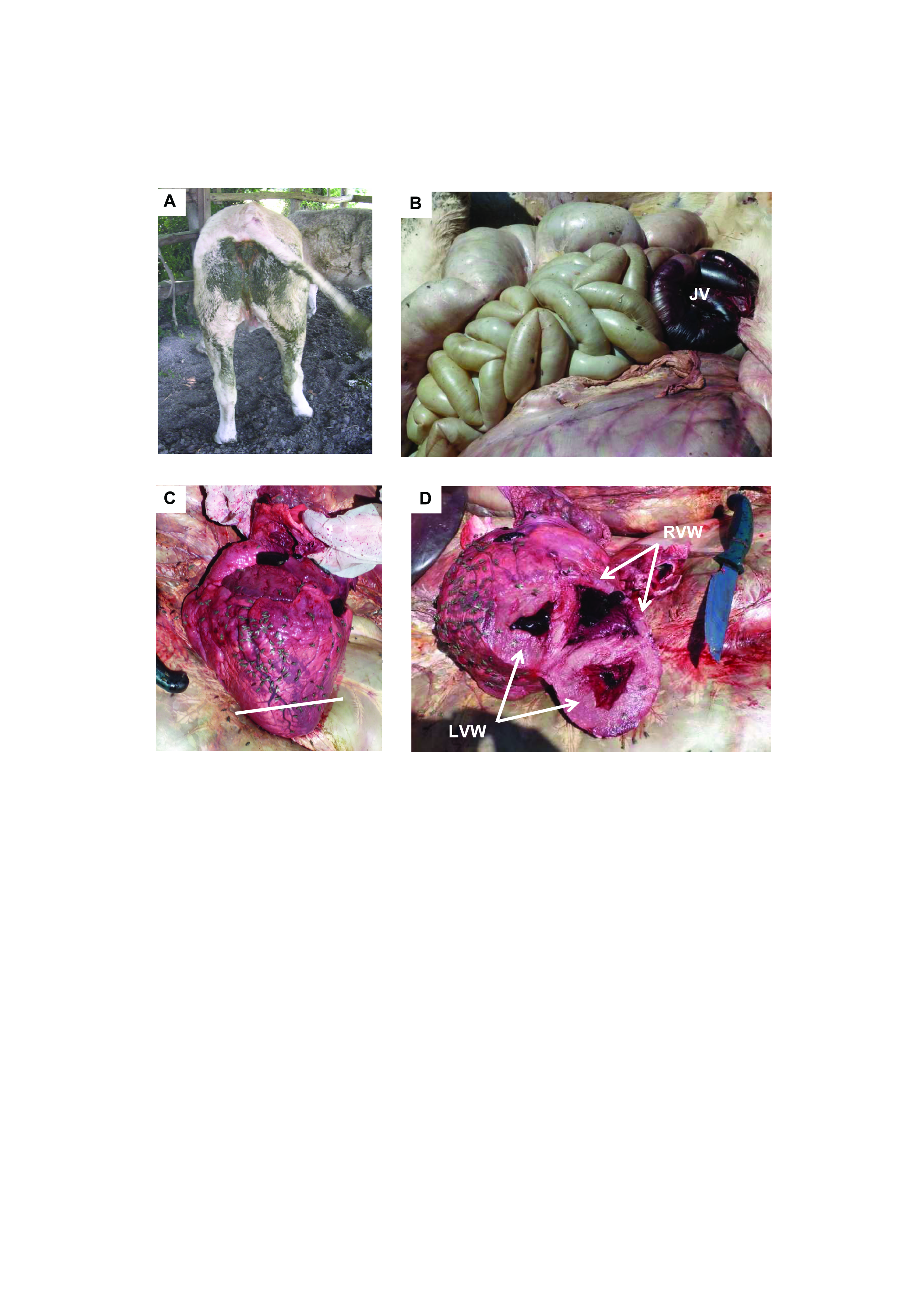

Supplement: Figure S1 — Additional illustrations of PMS clinical features. (A) Two-and-half-year old affected heifer displaying chronic diarrhea. (B) Autopsy of the abdominal cavity showing jejunal volvulus (JV) with focal hemorrhagic enteritis. (C) Heart of the same animal. White line indicates the section plane. (D) Transversal section of PMS heart showing ventricles in partially open position. RVW and LVW indicate respectively right ventricle wall and left ventricle wall. (TIF) [file pone.0049084.s001.tif]

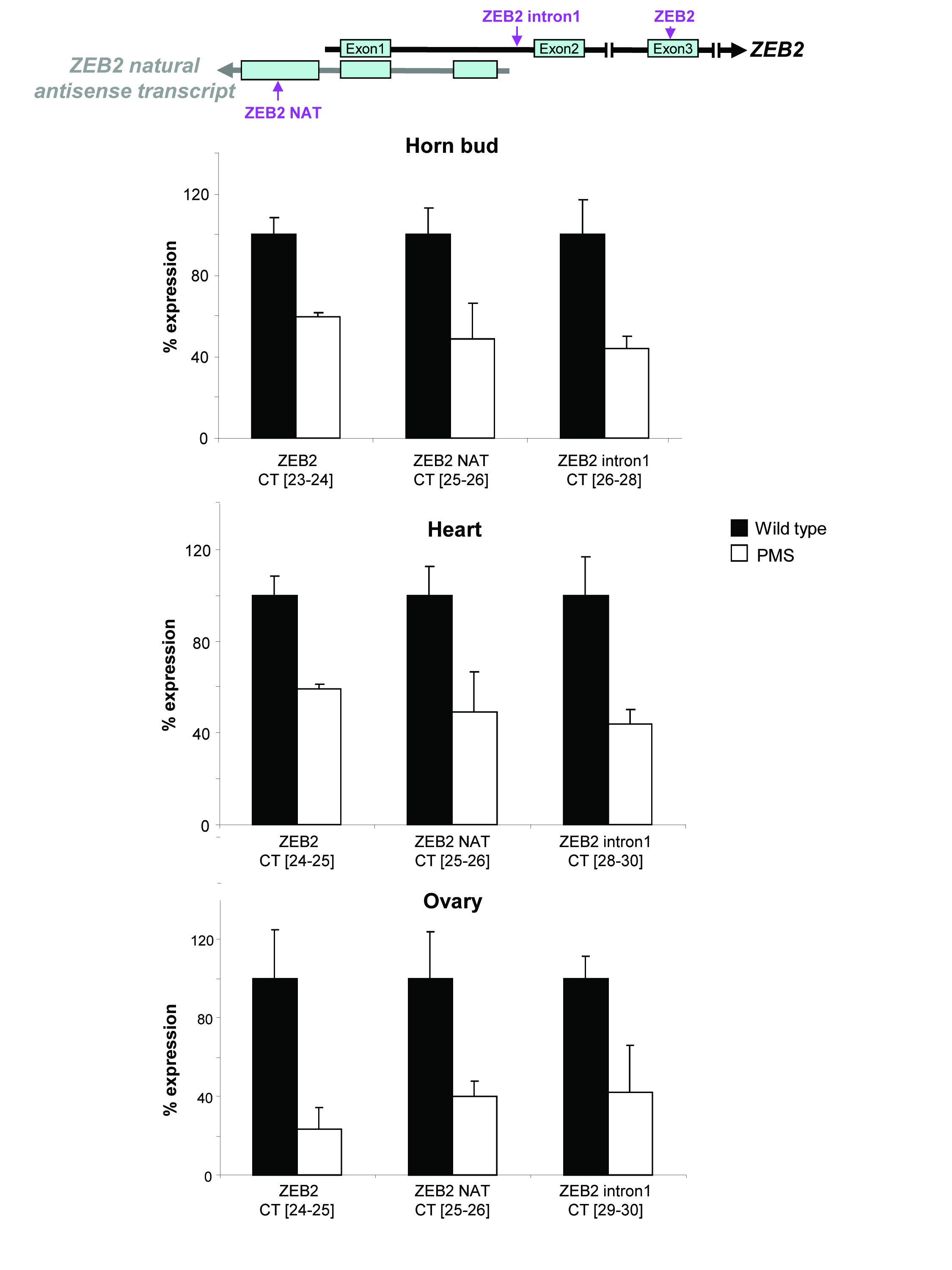

Supplement: Figure S2 — Analysis of ZEB2 expression regulation by its natural antisense transcript in different affected tissues at 90 dpc using real-time PCR. Localisation of PCR primers relatively to ZEB2 gene and its natural antisense transcript (NAT) are indicated by purple arrows. CT: Cycle Thresholds. Note the proportional reduction of ZEB2 exon 3, ZEB2 NAT and ZEB2 intron 1 RNA amounts in PMS versus wild type fetuses in the different organs studied. (TIF) [file pone.0049084.s002.tif]

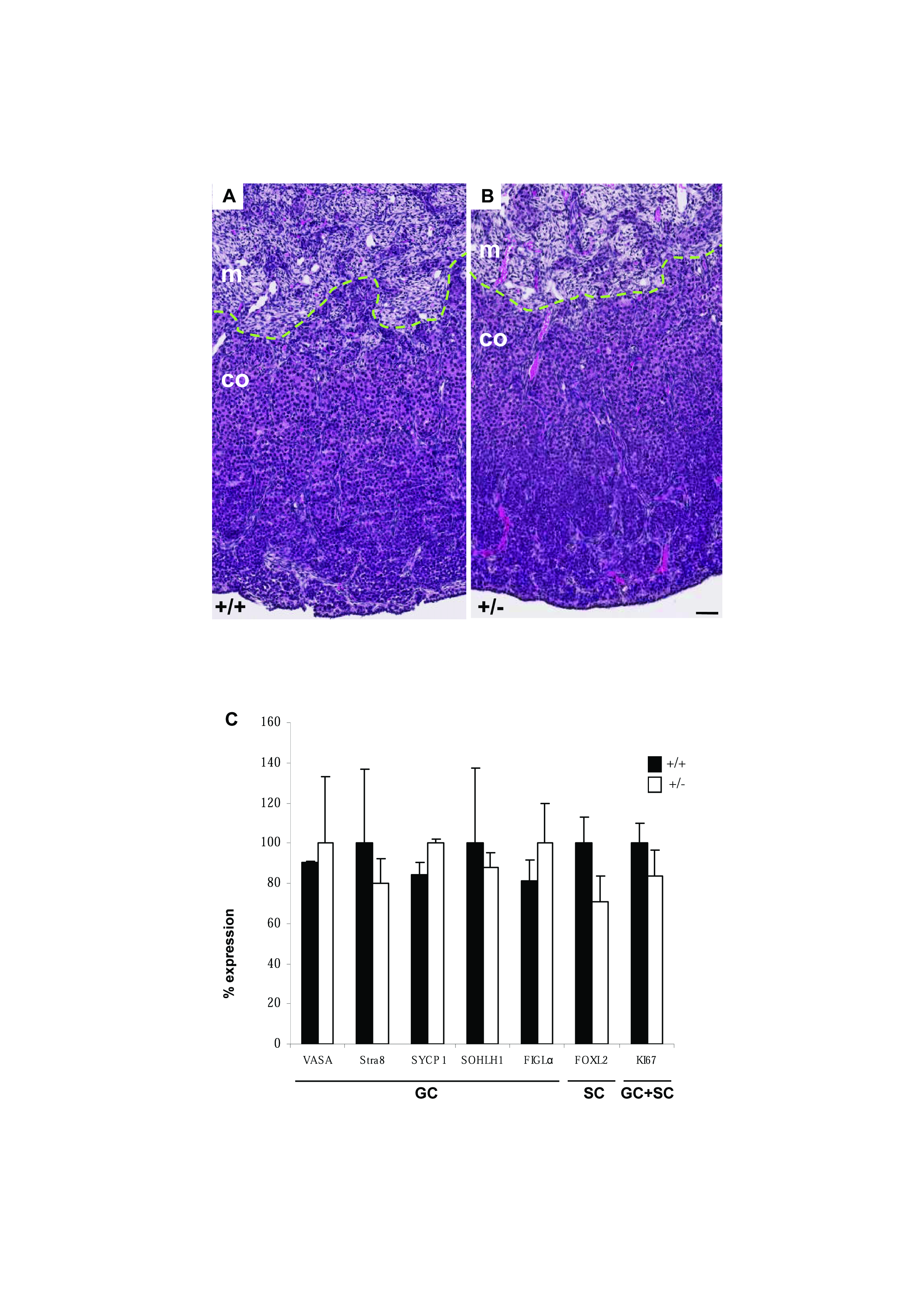

Supplement: Figure S3 — Histological and gene expression analyses of wild-type (+/+) and PMS (+/−) 90 dpc ovaries. Ovarian histology in +/+ (A) or +/− (B) fetuses at 90 dpc. The green dotted line shows the separation between the cortical (co) and the medulla (m) part of the ovaries. Scale bar represents 50 µm. (C), RT-PCR expression analyses of germ cells (GC) specific markers such as VASA and meiotic markers (STRA8 and SYCP1), or genes involved in follicle formation (SOHLH1 and FIGα), in wild type or mutant 90 dpc ovaries. The somatic cell (SC) marker FOXL2 and proliferation factor (KI67) were also studied. (TIF) [file pone.0049084.s003.tif]
