# Supplementary material for: A 3.7 Mb Deletion Encompassing ZEB2 Causes a Novel Polled and Multisystemic Syndrome in the Progeny of a Somatic Mosaic Bull
Source: PLoS One. 2012 Nov 9;7(11):e49084. doi: 10.1371/journal.pone.0049084 (PMC3494662; doi:10.1371/journal.pone.0049084)
Supplement: Table S1 — Primers used in RT-qPCR study. (DOC) [file pone.0049084.s004.doc]

| *Gene* | *Forward primer* | *Reverse primer* |
| --- | --- | --- |
| ACTB | CAGCAAGCAGGAGTACGATGAG | AAGGGTGTAACGCAGCTAACAGT |
| ARHGAP15 ex13 | ACAGCTTAGGGATTGTGTTTGGA | AGCTCGGCGATCTGGTTCT |
| FIGLα | AGGACGTGCAGTTGGTGCTG | CAGTGCCTTCAGTTTGGCCA |
| FOXL2 | CCGGCATCTACCAGTACATTATAGC | GCACTCGTTGAGGCTGAGGT |
| GTDC1 | CCTGTTGTATAACCGCACTCATGT | TCAGGCACCATGTCATCCAGTA |
| H2AFZ | GCGTATTACCCCTCGTCACTTG | CAGCAATTGTAGCCTTGATGAGA |
| Ki67 | CAGCATGGGATCCTCCAGAC | CCGAGTTTCACCACATCTGC |
| KYNU | AAGTGCTTTGACTTTCTCACTCAGAA | GTCCACAGCTCCCAGTGTCTCT |
| SOHLH1 | AGCATCTTCCCCGACTTCTT | CAGCCCAAACCCAGAATAAA |
| Stra8 | TGTTGAGGTGCACAGAGACGTT | GAACTCAAAGCCGGAGACCAT |
| SYCP1 | AAGCTTCCCATCAGCTAATCAG | TGCCTTTGGTAATGGTGTAGATAA |
| VASA | CGAGGGCTGGATATTGAAAA | TGCCAGTATTTCCACAACGA |
| YWHAZ | GGAGCCCGTAGGTCATCTTG | CTCGAGCCATCTGCTGTTTTT |
| ZEB2 | ACACGGGTTCTGAAACTGATGA | GGCACGCTAGCTGGACTTCT |
| ZEB2 NAT | AGAGAAACTTGGCGATCACG | GAGAGAGAGACCCTGAAACACG |
| ZEB2 intron1 | TCCTCATGGAACTTGAGTCG | AAAACCCTACCATCCTGACG |
